# Clinical and Surgical Outcomes of Shoulder Arthrodesis

**DOI:** 10.3390/jcm13164701

**Published:** 2024-08-10

**Authors:** Salar Sobhi, Kieran Bochat, Grant Booth, Andrew Mattin, Sheldon Moniz

**Affiliations:** 1 Department of Orthopaedic Surgery, The Orthopaedic Research Foundation of Western Australia (ORFWA), Murdoch, WA 6150, Australia; 2 Department of Orthopaedic Surgery, Royal Perth Hospital, Perth, WA 6000, Australia

**Keywords:** shoulder arthrodesis, glenohumeral joint arthrodesis

## Abstract

**Introduction:** Shoulder arthrodesis is typically seen as a salvage procedure with limited functional objectives. In appropriately selected patients, it may effectively alleviate pain, provide stable motion, and offer patient function satisfaction. However, there have been few reports on the outcomes following shoulder arthrodesis. **Methods:** A multicenter, retrospective chart review of patients undergoing shoulder arthrodesis between 2001 and 2023 in Western Australia was conducted. Clinical records and imaging were then reviewed to determine patient demographics. A cross-sectional analysis of Visual Analogue (VAS), Oxford Shoulder (OSS), and American Shoulder and Elbow Surgeons Shoulder (ASES) Scores, satisfaction and complication rates was conducted. **Results:** In total, 14 patients with a mean age of 39.5 years (range 22–52 years, 71% male) with a mean follow-up of 7.4 years (range 3 months–18 years) were identified. The most common indications for arthrodesis included osteoarthritis (8, 57%) and instability (6, 43%). Major contributory factors were recurrent seizures (5, 36%) and multiple surgeries (4, 29%). Radiographic union was observed in 13 (93%) patients. The mean VAS was 2.8 (range 0–7), mean OSS was 33.0 (range 23–42) and ASES score was 55.4 (range 37–82). In total, 11 patients (79%) reported being satisfied. Five (36%) patients returned to theatre for complications. **Conclusions**: In this patient series, shoulder arthrodesis demonstrates a notable efficacy in pain reduction, high satisfaction, acceptable function, and complication rates.

## 1. Introduction 

Shoulder joint arthrodesis is generally considered a salvage procedure that aims to provide a painless and stable shoulder position to allow function using scapulothoracic, elbow, and hand motion [1]. Shoulder arthrodesis has been employed less in recent years with improved techniques and prostheses relating to reverse shoulder arthroplasty. Common indications for shoulder arthrodesis include brachial plexopathies, refractory instability, significant glenohumeral bone loss, deltoid and rotator cuff deficiency, and chronic infections [2,3]. The rotator cuff muscles work not only as a motion actuator (abduction or external and internal rotations) but also as a shoulder stabilizer. In the case of untreated rotator cuff tears, the humeral head can move upward and rub against the acromion, resulting in glenohumeral joint arthropathy.

Shoulder arthrodesis can be achieved using intra-articular and extra-articular techniques [1]. Most commonly, compression screws or plate fixation are employed for this procedure [4,5,6]. Despite the unique set of indications and goals of this procedure, shoulder arthrodesis is not commonly performed as revision shoulder arthroplasty has equivalent relief rates and improved functional outcomes. Shoulder arthrodesis can still be an option for patients who have undergone multiple failed revision arthroplasties or those with glenohumeral arthritis accompanied by rotator cuff and deltoid muscle dysfunction, as it may offer a more predictable outcome [7].

Despite improvements in modern materials and techniques, shoulder arthrodesis is associated with complication rates as high as 43% [1,8,9]. These include but are not limited to component malposition, infection and non-union. Outcomes following shoulder arthrodesis are seldom reported in the literature. This is due to the heterogeneity of patient indications, variations in techniques and arthrodesis methods, and lack of long-term follow-up. Few studies exist investigating shoulder arthrodesis in a larger cohort of mixed indications, and many of these do not report outcomes using standardised function scores [1].

This retrospective review aims to report the subjective and objective short- and long-term results of shoulder arthrodesis for 14 patients.

## 2. Methods

### 2.1. Patients

This was a multi-centre, multi-surgeon retrospective review of patients who underwent shoulder arthrodesis between 2001 and 2023 in Western Australia. The procedures were performed in three high-volume tertiary orthopaedic centres by four orthopaedic surgeons with a subspecialty interest in shoulder surgery.

Inclusion criteria—patients who underwent shoulder arthrodesis during the aforementioned time period and were above 18 years of age at the time of data collection for this study.

Exclusion criteria—patients with mental compromise (i.e., currently being treated for a psychiatric disorder, senile dementia, Alzheimer’s disease, presence of alcohol and/or substance abuse), or an inability to provide informed consent.

This study was conducted according to the guidelines of the Declaration of Helsinki and approved by the South Metropolitan Health Service Governance Evidence Knowledge Outcomes (GEKO) Committee (GEKO approval No. 42649). All participants provided informed consent for data collection for this study.

### 2.2. Operative Technique and Rehabilitation

**Figure 1:**
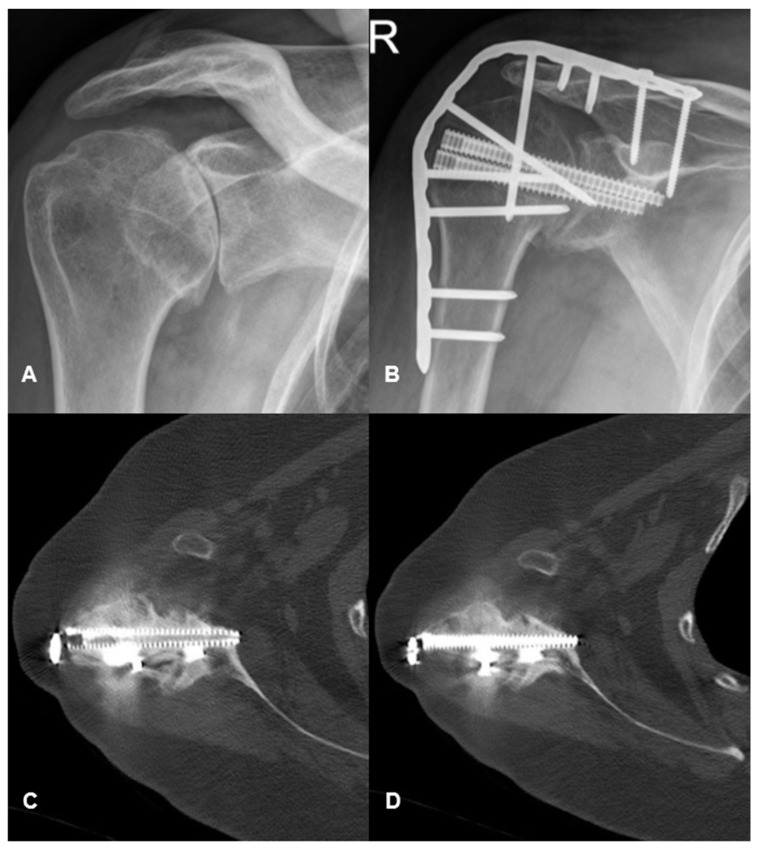
(**A**) shows an anteroposterior radiograph of the right shoulder demonstrating evidence of osteoarthritis in a patient with recurrent septic arthritis. (**B**) shows the same patient undergoing shoulder arthrodesis with screw and plate constructs. (**C**,**D**) are post-operative axial computed tomography scans demonstrating bridging callus and arthrodesis of the shoulder joint.

All patients underwent a general anaesthetic, were in a supine position with a beach chair, and received perioperative antibiotics. An S-shaped skin incision beginning over the scapular spine, traversing anteriorly over the acromion, and extending down the anterolateral aspect of the arm was employed in all cases. The patient’s passive ability to move their hand to their mouth and face intra-operatively was used as the arthrodesis position, which was generally 30° abduction, 30° internal rotation, and 30° forward flexion. Plate and screw constructs were used in all cases (Figure 1).

All patients underwent post-operative multimodal analgesia and input from the hospital’s acute pain service. All patients underwent sling immobilisation for at least 6 weeks post-operatively. All patients underwent serial clinical and radiological review post-operatively to assess efficacy of the arthrodesis (Figure 1).

### 2.3. Baseline Demographics

A retrospective review of patient’s medical records was conducted. Baseline demographics including age, sex, hand dominance, side of surgery, occupation, date of surgery, and indications for arthrodesis, past medical history including smoking status, drug and opioid use were collected.

### 2.4. Assessment of Clinical Outcomes

In this cross-sectional study, patients were interviewed and had their clinical outcomes assessed using the Visual Analogue Scale (VAS), Oxford Shoulder Score (OSS), and American Shoulder and Elbow Surgeons Standardised Shoulder (ASES) scores. Patient were also asked if they were satisfied with their arthrodesis.

Visual Analogue Scale (VAS)—A validated, subjective measure for acute and chronic pain. Patient scores were recorded by making a handwritten mark on a 10 cm line that represents a continuum between “no pain” and “worst pain” [10].

Oxford Shoulder Score (OSS)—A validated scoring system used to assess the degree of pain and disability caused by shoulder pathology [11]. It contains 12 items, each with 5 potential answers. A mark between 1 (best/fewest symptoms) and 5 (worst/most severe) is awarded to correspond to the patient’s symptoms. The combined total gives a minimum score of 12 and a maximum of 60. A higher score implies a greater degree of disability [12].

American Shoulder and Elbow Surgeons Standardised Shoulder (ASES) Score—A validated joint-specific score used to assess shoulder pain and function. The ASES is a 100-point scale that assess the following three sections: pain, instability, and activities of daily living. The combined total gives a minimum score of 0 and a maximum of 100, of which 50 is attributed to pain. A high score implied a greater degree of function, stability, and less pain [13].

Additionally, complications that required patients to return to the operating room were noted and analysed.

### 2.5. Statistical Analyses

Given the cross-sectional nature of this study, proportions were presented as percentages (%). Averages of functional outcomes VAS, OSS and ASES were presented as means and range.

## 3. Results

### 3.1. Patient Characteristics

Table 1 shows the demographic data, complications, and functional results of all patients. A total of 14 patients with a mean age of 39.5 years (range 22–52 years) with a mean follow-up of 7.4 years (range 3 months–18 years) were identified in our multi-hospital database. Most cases (10 out of 14, 71%) undergoing shoulder arthrodesis were male, involved their dominant limb (9 out of 14, 64%), and were unemployed (8 out of 14, 57%). Furthermore, 4 (29%) patients experienced opioid dependence and 8 (57%) were active smokers at the time of review.

The most common indication for shoulder arthrodesis was osteoarthritis (8 out of 14, 57%), followed by instability (6 out of 14, 43%). The major contributory factors for performing shoulder arthrodesis were recurrent seizures (5 out of 14, 36%) and multiple failed surgeries (4 out of 14, 29%). A total of 4 out of 14 patients (29%) in this cohort had a previous or current history of intravenous drug use (IVDU).

### 3.2. Outcomes

Out of the 14 patients, 13 (93%) achieved radiographic union. The mean pain score measured through the VAS was 2.8 (range 0–7). The mean OSS of the cohort was 33.0 (range 23–42). The mean ASES score was 55.4 (range 37–82). A total of 11 out of 14 patients (79%) reported being satisfied with their procedure at the time of reporting.

### 3.3. Complications

**Figure 2:**
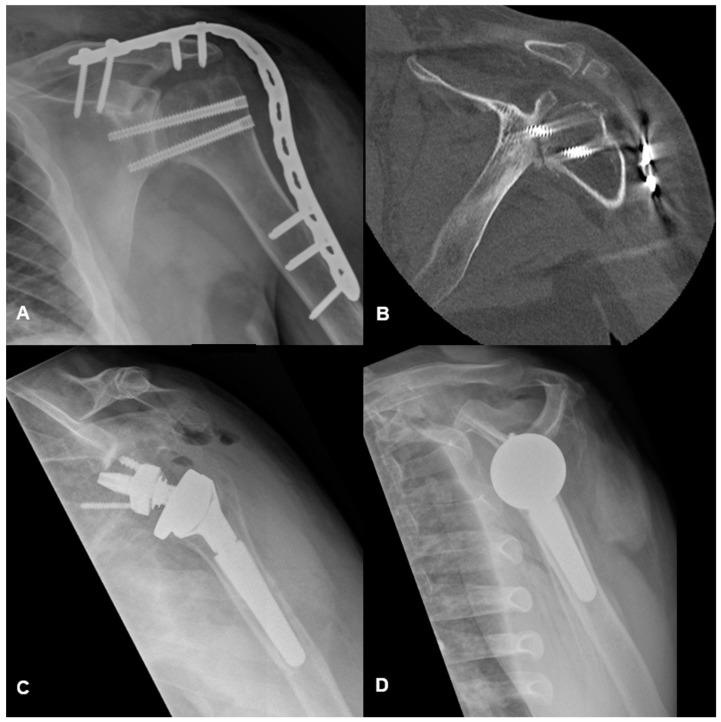
(**A**) shows an anteroposterior radiograph of the left shoulder of a patient with ongoing pain and mobility 4 months post-shoulder arthrodesis for osteoarthritis and recurrent seizures. (**B**) shows a post-operative axial computed tomography scan demonstrating lack of bridging callus formation. (**C**,**D**) are post-operative radiographs of the same patient undergoing reverse total shoulder arthroplasty after being seizure-free for 12 months.

A total of five (36%) patients returned to the operating room for complications. One patient required revision arthrodesis due to malunion. Two required metalwork removal for infection and one for prominent metalwork. One patient who underwent shoulder arthrodesis for osteoarthritis secondary to recurrent seizures underwent conversion to reverse total shoulder replacement due to ongoing pain and lack of callus formation at four months post-arthrodesis (Figure 2). Lastly, one patient is awaiting elective plate removal.

## 4. Discussion

To date, our cross-sectional study is one of the largest to contribute to the small body of literature available relating to outcomes following shoulder arthrodesis with plate and screw fixation. In this cohort, shoulder arthrodesis was predominantly performed in young males with osteoarthritis or instability. Major contributory factors to having this procedure included recurrent seizures, multiple surgeries, and history of IVDU. Our results showed acceptable functional scores, high union (93%), and satisfaction rates (79%). The post-operative complication rate requiring return to the operating room for this procedure was 36%.

The indication for shoulder arthrodesis has decreased in the last few years due to recent advances in surgical techniques and arthroplasty. However, it is still considered a salvage procedure in specific patients with paralytic or pseudoparalytic shoulders, chronic refractory instability, infection, failed arthroplasty, or after tumour resection [14]. Despite the limited function achieved with this procedure, it improves pain and patients were generally satisfied in the context of their significant co-morbidities in this patient series.

### 4.1. Outcomes

The objective outcomes of shoulder arthrodesis in our cohort demonstrated acceptable scores comparable to those undergoing shoulder arthroplasty and previous arthrodesis cohorts in the literature. Given the inability for shoulder arthrodesis patients to elevate their arm to 90°, only the VAS, OSS and ASES shoulder index were employed in this study. The mean OSS of 33.0 (range 23–42) found in our cohort is considered an acceptable result when compared with patients undergoing shoulder arthroplasty, where a patient-acceptable symptom state (PASS) has been defined as a score above 29 Similarly [15], our mean VAS of 2.8 (range 0–7) and ASES of 55.4 (range 37–82) were similar to Dimmen et al.’s (2007) cohort of 18 shoulder arthrodesis patients who were followed-up between 3 and 15 years who and who had mean VAS and ASES scores of 1.6 (range 0–8) and 59 (15–95), respectively^4^ Conversely, the mean ASES score of our cohort was significantly lower than the acceptable PASS estimate of 76 for patients undergoing shoulder arthroplasty [16]. This can be explained as the ASES has many components that assess the ability to perform overhead activities that are not possible following arthrodesis, thus resulting in lower scores. Additionally, it is known that shoulder arthroplasty results in a superior range of motion and therefore function.

### 4.2. Satisfaction

Similarly, our high satisfaction rate of 79% is consistent with previously published literature. Górecki et al.’s (2021) systematic review of 294 patients undergoing shoulder arthrodesis for brachial plexus injury reported a subjective satisfaction rate of 82% (range 59–100%) and similarly, Dimmen et al.’s (2007) cohort reported a satisfaction rate of 83% relative to their pre-operative condition [4,8]. Furthermore, there is no difference in satisfaction when comparing plate and screw stabilisation in the literature [8]. Given the chronic pain, instability and multiple surgeries seen in this cohort, a definitive procedure to help address these issues allowing an acceptable level of pain and function, with the ability to perform basic everyday activities such as eating or personal hygiene, may attribute to this high satisfaction rate.

### 4.3. Complications

Our reported complication rate requiring return to the operating room was 36%. Additionally, another two patients in the cohort had planned metalwork removal. Recent studies show that the incidence of all complications following shoulder arthrodesis is 28%, and has been reported as high as 43% [8].Early complications are infrequent and include surgical site infection, breakdown of skin and haematoma formation^8^ Later complications include non-union, being as high as 20% in some series, infection, malunion, periprosthetic fracture, hardware prominence and irritation.

Ruhmann et al. (2002) showed that 9% of patients had non-union [17]. This was followed by another series in 2005 that showed that the union rate was high in plate compared to screw arthrodesis [18]. Dimmen et al. (2007) demonstrated a union rate of 89% with 2 out of 18 patients demonstrating asymptomatic partial fusion (glenohumeral or acromiohumeral) radiographically [4]. This was similar to our radiographic union rate of 93%, with our single case of non-union converted to a reverse total shoulder arthroplasty at 4 months post-arthrodesis.

The infection rate following shoulder arthrodesis is reported to range between 0 and 14%. In our cohort, where all patients received perioperative antibiotics, we demonstrated a 14% infection rate (2 out of 14 patients), requiring removal of metalwork and washout. Furthermore, periprosthetic fracture rates following shoulder arthrodesis are reported to be up to 11% in the literature. A combination of an immobile shoulder joint, osteopenia and development of a stress riser due to the plate can result in periprosthetic fracture. Cofield and Briggs (1979) had eight fractures in their series of 71 patients (11%), whilst no fractures were reported in our cohort or in Richards et al. (1988) and Stark et al.’s (1991) cohorts [2,5,6]. Lastly, we had one case of plate removal due to irritation and one planned elective plate removal. Several studies have demonstrated the need for hardware removal after union for symptomatic plates and/or screws [5,6,9]. Most commonly, irritation occurs over the scapular spine and acromion due to less soft tissue coverage [9].

### 4.4. Limitations

This study must be viewed considering its limitations. Firstly, data collection was retrospective and functional outcomes were obtained cross-sectionally. Therefore, we are unable to ascertain the outcomes of shoulder arthrodesis over time. Secondly, the single-arm nature of this study makes interpretation of outcomes difficult, given there is no cohort for comparison. Thirdly, we were unable to collect data on certain outcomes such as range of motion, which makes it difficult to ascertain what level of function is possible in our cohort. Lastly, the wide range of follow-up (3 months to 18 years) makes it difficult to accurately quote a complication rate; however, it is historically known that shoulder arthrodesis has frequent complications.

## 5. Conclusions

In our cross-sectional review of 14 patients identified as suitable candidates for salvage procedures addressing diverse shoulder pathologies, shoulder arthrodesis demonstrated notable efficacy in pain reduction. Furthermore, it has the potential to attain a satisfactory level of function and subjective contentment in the best-case scenario.

## Figures and Tables

**Table 1:** Outcomes of patients following shoulder arthrodesis.

A	B	C	D	E	F	G	H	I	J	K	L	M	N
35	F	N	Y	N	N	Humeral head AVN Axillary nerve palsy	Multiple surgeries	18	3	38	40	Nil	Y
35	M	N	N	Y	N	Humeral head AVN	Multiple surgeries	7	7	41	30	Nil	N
38	M	Y	N	N	Y	GHJ OA Rotator cuff insufficiency	IVDU	5	2	68	36	Malunion, required revision	Y
45	M	N	N	N	N	GHJ OA Axillary nerve palsy Instability	Seizures	5	2	45	41	Washout, plate removal for infection	Y
27	M	Y	Y	Y	Y	GHJ OA Instability	Multiple surgeries	4	2	62	38	Removal of plate due to irritation, CRPS	Y
33	M	Y	Y	N	N	Erb’s palsy GHJ OA		8	0	56	23	Nil	Y
22	F	Y	Y	N	N	Chronic anterior dislocation	Seizures	7	4	42	33	Nil	Y
42	M	N	N	N	Y	Instability	Seizures, IVDU, TBI	11	0	82	26	Nil	Y
48	M	Y	Y	N	Y	GHJ OA	IVDU	10	0	70	23	Nil	Y
43	M	Y	Y	N	Y	GHJ OA Instability		8	4	58	27	Nil	Y
50	F	Y	N	Y	Y	GHJ OA	RA, previous SA	9	4	56	32	Infection, removal of metalwork	Y
47	M	Y	N	Y	Y	Humeral head AVN	IVDU, schizophrenia	10	0	48	29	Planned plate removal	N
36	F	N	N	N	N	Instability	Seizures Multiple surgeries	9 m	4	73	42	Nil	Y
52	M	Y	N	N	Y	GHJ OA	Seizures	4 m	7	37	42	Lack of callus. Converted to rTSR.	N

A—age at operation; B—sex; C—dominant limb; D—employed; E—opioid dependence; F—smoker; G—indication; H—contributory factors; I—follow-up (years); J—visual analogue score; K—ASES score; L—Oxford shoulder score; M—complications; N—satisfied.

## Data Availability

The data presented in this study are available on request from the corresponding author.
